# Efficacy of radiotherapy combined with systemic therapy for retroperitoneal lymph node metastasis in upper tract urothelial carcinoma(UTUC) patients after radical nephroureterectomy

**DOI:** 10.1186/s12957-025-03943-7

**Published:** 2025-11-11

**Authors:** Xiaoying Li, Xian-Shu Gao, Hongzhen Li, Mingwei Ma, Siming Li, Bing Wang, Zheng Zhang, Chunru Xu, Qi Tang, Zihao Tao, Chuanliang Cui, Dian Wang, Xuesong Li

**Affiliations:** 1 Department of Radiation Oncology, Peking University First Hospital, No.7 Xishiku Street, XichengBeijing, 100034 District China; 2 Department of UrologyInstitution of UrologyBeijing Key Laboratory of Urogenital Diseases (Male) Molecular Diagnosis and Treatment Center, Peking University First HospitalPeking UniversityNational Urological Cancer Center, Beijing, 100034 China; 3 Department of Renal Cancer and Melanoma, Key Laboratory of Carcinogenesis and Translational Research, Ministry of Education/Beijing), Peking University Cancer Hospital & Institute, Beijing, China; 4 Department of Urology, Peking University First Hospital- Miyun Hospital, Beijing, 101500 China; 5 Department of Radiation Oncology, Rush University Medical Center, Chicago, IL USA

**Keywords:** Upper tract urothelial carcinoma, Retroperitoneal lymph node metastasis, Radiotherapy, Prognosis, Adverse effect

## Abstract

**Background:**

Retroperitoneal lymph node metastasis (rLNM) of upper tract urothelial carcinoma (UTUC) after radical nephroureterectomy (RNU) represents a distinct metastatic state. Due to the unclear efficacy and side effects of radiotherapy in the treatment of these patients.This study aimed to assess the efficacy and adverse effects of radiotherapy combined with systemic therapy in rLNM patients after RNU.

**Methods:**

A total of 114 UTUC patients with retroperitoneal LNM after RNU were prospectively enrolled database with retroperitoneal LNM after RNU. Patients were classified by initial treatment type: (1) radiation therapy, (2) systemic therapy or (3) combination therapy. Cox proportional hazard models were used to assess the factors associated with progression free survival (PFS) and overall survival (OS). Survival curves for each group were generated and compared via the Kaplan‒Meier method.

**Results:**

A total of 114 rLNM patients with a median recurrence-free interval of 8 months after surgery (range: 1–108) were analyzed. During the median follow-up of 22 months (range: 3–81 months), 60 patients (58.8%) developed distant metastases. The para-aortic region was the most frequent site of recurrence. The recurrence rate in this region was significantly higher in patients with renal pelvic and proximal ureteral tumors (UTUCs) than in those with middle and distal UTUCs (*P* = 0.046). The common iliac region was the second most common recurrence site. Compared with renal pelvic tumors, ureteral tumors were more likely to recur in the common iliac region (*P* = 0.001). We delineated the radiotherapy clinical target volume (CTV) on the basis of primary tumor site; only 3 patients developed in-field recurrence during follow-up.The 2-year PFS rates for the combination therapy, radiation therapy and systemic therapy groups were 65.7%, 21.1% and 20.2%, respectively (*P* < 0.001). The 2-year OS rates for the combination therapy, radiation therapy and systemic therapy groups were 87.9%, 48.1% and 45.6%, respectively (*P* < 0.001). Multivariate analysis revealed that combination therapy,radiotherapy beginning within 3 months after recurrence and maintenance therapy after radiation were independent predictors of PFS. Maintenance therapy after radiation and distant metastasis were significant predictors of OS. Among 55 patients, 1 patient (1.8%) experienced acute grade 3 adverse events during combination therapy.

**Conclusions:**

Systemic therapy and radiotherapy improved PFS compared with either therapy type alone in rLNM UTUC patients.The majority of patients experiencing only grade 1–2 gastrointestinal reactions.

**Supplementary Information:**

The online version contains supplementary material available at 10.1186/s12957-025-03943-7.

## Introduction

The management of advanced upper tract urothelial carcinoma (UTUC) continues to pose significant challenges. Following radical nephroureterectomy (RNU), these patients have a dismal prognosis, with 5-year survival rates ranging from 30 to 50%[1]. Although adjuvant chemotherapy (ACT) confers survival benefits in high-risk UTUC patients, approximately one-third (29%) of these patients develop metastatic progression within 3 years after surgery[2].

These patients often present with chemotherapy resistance or become ineligible for platinum-based chemotherapy (PBC) secondary to post-RNU renal insufficiency[3]. Notably, even among platinum-eligible patients with metastatic disease, long-term outcomes remain unsatisfactory[4].Immunotherapy represents an alternative strategy; however, PD-L1-negative UTUC patients derive limited benefit, with suboptimal median progression-free survival (PFS)[5].

In our previous retrospective study, the majority of postoperative recurrences in UTUC patients occurred in the retroperitoneal lymph nodes[6].Notably, patients in the lymph node-only metastasis subgroup derived greater benefit from the EV-302 regimen than patients with visceral metastases did[7].According to the European Association of Urology (EAU) guidelines,UTUC classified clinical locoregional LN metastases(cN +) as metastatic disease.Patients with cN + disease should be treated via template-based lymph node dissection (LND) during RNU for select patients—particularly systemic therapy responders[4].

However, standardized template lymph node dissection is technically challenging in UTUC. Urothelial carcinoma is a relatively radiosensitive tumor. Bladder cancer designates pelvic lymph node (LN) involvement as N1–3 disease[4], chemoradiotherapy as a consolidative modality improves both overall survival (OS) and PFS in patients with pelvic or retroperitoneal LN metastases[8]. According to the European Association of Urology (EAU) guidelines, radiotherapy, chemotherapy, and possibly surgery, either alone or in combination, should be recommended as options the treatment of local recurrence in muscle-invasive bladder cancer (MIBC) patients after radical cystectomy (RC)[9].

However, the role of consolidative radiotherapy in UTUC patients with retroperitoneal lymph node metastasis (LNM) remains investigational. This prospective observational study focused on identifying the optimal treatment strategy for clinically node-positive (cN +) UTUC patients after radical nephroureterectomy (RNU). In particular, we assessed the efficacy and safety of radiotherapy combined with systemic therapy compared with current standard treatments.

## Materials and methods

### Study population

Patient data were retrieved from a prospective dataset collected from November 2017 to April 2024, and UTUC patients with retroperitoneal LNM after RUN were enrolled from three hospitals in China. Lymph node dissection (LND) was performed when LNM was suspected during the preoperative evaluation or when enlarged lymph nodes were identified during surgery. The inclusion criteria for the study were as follows: (1) pathological diagnosis of urothelial carcinoma of the renal pelvis or ureter, (2) pathological stage T1-4aN0-2M0 with radical nephroureterectomy, and (3) any enlarged lymph nodes with FDG avidity by positron emission tomography (PET) or new enlarged lymph nodes with short axes diameter of 0.6 cm on CT and/or MR images during regular follow-up. The exclusion criteria were as follows: (1) presence of a previous second primary cancers (except for bladder tumors), (2) nonradical nephroureterectomy, or (3) radical nephroureterectomy with distant metastasis.

The histological diagnosis was based on the 2004 World Health Organization (WHO) classification. Moreover, the tumors were staged according to the American Joint Committee on Cancer (AJCC) staging system, 8th edition. The primary tumors were classified into four groups according to the tumor site: renal pelvis tumor, proximal ureter tumor, middle ureter tumor and distal ureter tumor.

## Systemic therapy

Since enfortumab vedotin (EV) only received approval from China's National Medical Products Administration (NMPA) in 2025, none of the patients in this study were treated with EV. The first-line treatment for metastatic UTUC is cisplatin-based combination chemotherapy. For patients who are unfit for cisplatin-based combination chemotherapy, carboplatin-based chemotherapy is recommended as an alternative.

Patients meeting any of the following criteria are considered unfit for chemotherapy: (1) World Health Organization (WHO) or Eastern Cooperative Oncology Group performance status (ECOG PS) ≥ 2; (2) creatinine clearance (CrCl) < 60 mL/min; (3) grade ≥ 2 hearing loss; (4) grade ≥ 2 peripheral neuropathy; and (5) New York Heart Association (NYHA) class ≥ III heart failure[10]. Immunotherapy is an alternative choice for patients who are not eligible for chemotherapy. Another option is disitamab vedotin (DV, RC48-ADC), a novel humanized antihuman epidermal growth factor receptor 2 (HER2) antibody conjugated with monomethyl auristatin E that was approved by the NMPA for use in patients with HER2-positive (immunohistochemistry 3 + or 2 +) metastatic urothelial carcinoma (UC) refractory to standard or regular therapies[11].

## Radiotherapy

All patients were required to undergo computed tomography (CT) from the 10th thoracic vertebral body to the inguinal lymph node area in the supine position with a 5-mm slice thickness to assist in tumor localization for radiotherapy planning. The treating radiation oncologist determined the scope of radiotherapy by contouring the gross target volume (GTV) and clinical target volume (CTV)[6]. Metastatic lymph nodes were defined as the gross tumor volume (GTV).The CTV included the lymph node region and differed by primary tumor site (Table 2). The pelvic and abdominal recurrent lymph node regions include the para-aortic, common iliac, external iliac, and internal iliac regions[12]. Para-aortic lymph nodes were further divided into three subgroups: those to the left of the aorta (left para-aortic or LPA nodes), those between the aorta and the inferior vena cava (aortocaval or AC nodes), and those to the right of the inferior vena cava (right paracaval or RPC nodes). A linear accelerator with kilovoltage cone-beam computed tomography was utilized for the radiation process, and treatment planning was performed using either the Medtronic Monaco or Varian Eclipse inverse intensity-modulated radiotherapy planning systems.The recommended dose was 60–62.5 Gy/25 fractions for the GTV and 45–50 Gy/25 fractions for the CTV.Dose constraints for organs at risk (OARs) were implemented as follows: Intestinal tract: Dmax < 52 Gy;Kidney:Mean dose < 15 Gy;V20 < 30%;V30 < 20%;Spinal cord: Dmax < 42 Gy.

## Follow-up and outcome measures

Abdominopelvic CT/MRI and thoracic CT were performed every 3 months, whereas cervical ultrasound and bone scans were conducted at 6-month intervals. In this study, bladder or contralateral recurrence was not considered a tumor progression event. The primary endpoint was PFS after rLNM. The secondary endpoints included OS after rLNM and the incidence of acute and late toxicities (CTCAE v5.0).

## Statistical analysis

Baseline characteristics were compared using the Mann‒Whitney U test for nonnormally distributed continuous variables and the χ^2^ test (or Fisher's exact test when expected cell counts < 5) for categorical variables, as appropriate. Univariate Cox regression was first performed to screen potential prognostic factors (inclusion threshold: *P* < 0.10). Significant variables were subsequently entered into multivariate Cox proportional hazards models with backward elimination (retention threshold: *P* < 0.05) to identify independent predictors of OS and PFS. Survival curves were generated and compared via the Kaplan‒Meier method with log-rank test. All analyses were conducted using SPSS 22.0 (IBM Corp.). A two-sided α level of 0.05 was considered to indicate statistical significance.

## Results

### Patient characteristics

**Fig. 1:**
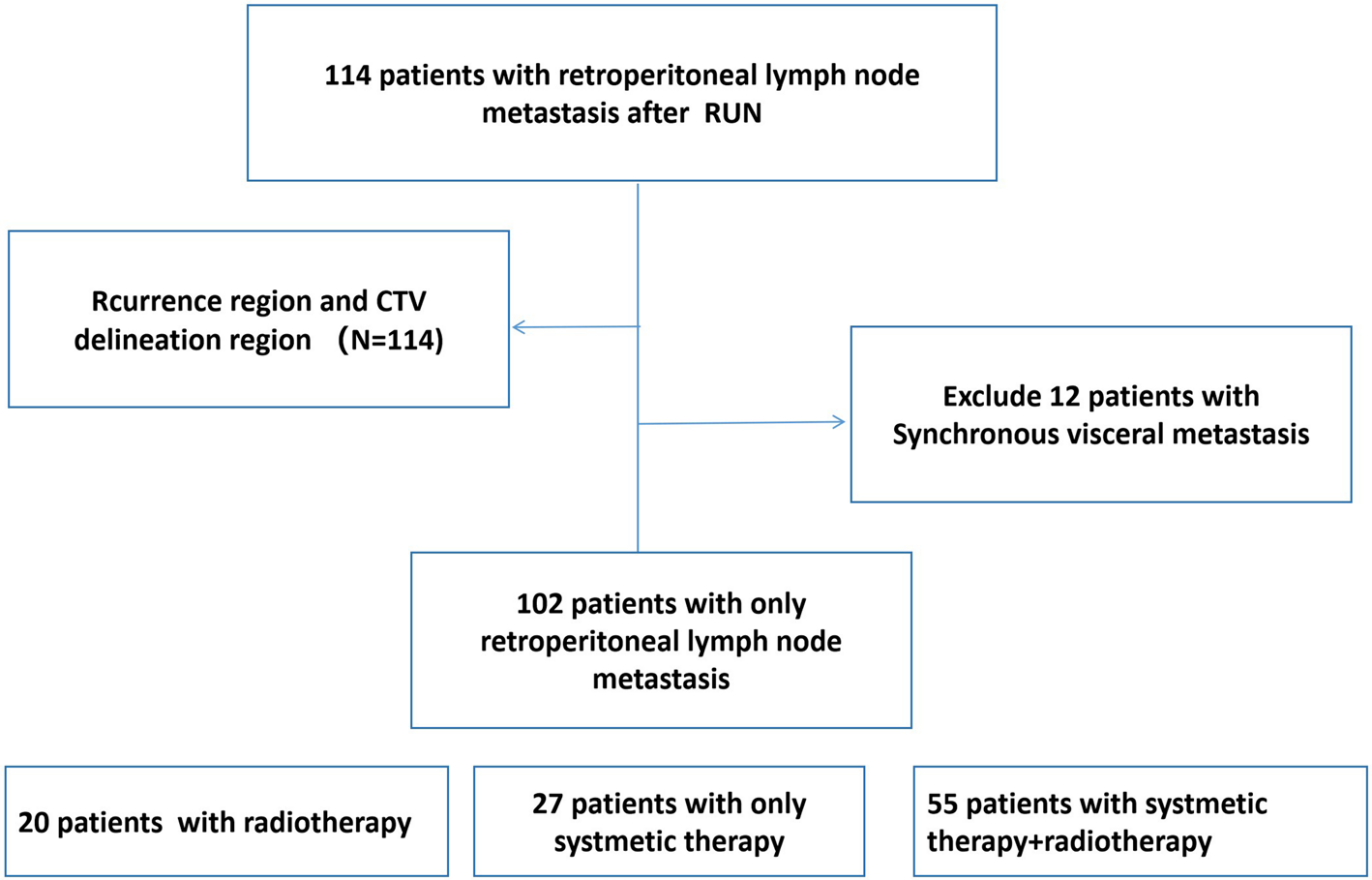
Patient disposition flow diagram

A total of 114 patients who underwent radical nephroureterectomy (RNU) and developed abdominal or pelvic recurrence during the study period were included (Table 1, Fig. 1). The median age was 55 years (range: 30–76 years). 19 patients in our study received postoperative adjuvant chemotherapy. The majority of recurrence events (90.35%, 103/114) occurred within 2 years after RNU, and the median recurrence-free interval was 8 months (range: 1–108 months). PET‒CT confirmed recurrence in 64.04% (73/114) of the patients. Among recurrence events, 302 recurrence lymph nodes were identified, with a median of 2 recurrence lymph nodes per patient (range: 1–11). For patients with LNR, the median number of involved lymph node regions was 1 (range: 1–3; Supplementary Table 1).

**Table 1:** Characteristics of 114 UTUC patients with positive lymph nodes

Characters	All patients *N* = 114	Renal Pelvis *N* = 32	Proximal ureter *N* = 10	Middle ureter *N* = 25	Distal ureter *N* = 47	*P* value
Age group						
< 70	61(53.5%)	17(53.1%)	5(50.0%)	14(56.0%)	25(53.2%)	0.990
≥ 70	53(46.5%)	15(46.9%)	5(50.0%)	11(44.0%)	22(46.8%)	
Gender			0			
Female	59(51.8%)	18(56.3%)	9(90.0%)	11(44.0%)	21(44.7%)	0.051
Male	55(48.2%)	14(43.7%)	1(10.0%)	14(56.0%)	26(55.3%)	
Multifocal tumor						0.140
No	93(81.6%)	25(78.1%)	6(60.0%)	20(80.0%)	42(89.4%)	
Yes	21(18.4%)	7(21.9%)	4(40.0%)	5(20.0%)	5(10.6%)	
Tumor grade						0.607
G2	26(22.8%)	9(28.1%)	2(20.0%)	7(28.0%)	8(17.0%)	
G3	88(77.2%)	23(71.9%)	8(80.0%)	18(72.0%)	39(83.0%)	
T stage						0.065
T1	10(8.8%)	2(6.3%)	1(10.0.0%)	3(12.0%)	4(8.5%)	
T2	32(28.1%)	5(15.6%)	0(0%)	7(28.0%)	20(42.6%)	
T3	66(57.9%)	22(68.8%)	8(80.0%)	14(56.0%)	22(46.8%)	
T4	6(5.3%)	3(9.4%)	1(10.0%)	1(4.0%)	1(2.1%)	
LNM						0.081
N0	35(30.7%)	7(21.9%)	4(40.0%)	5(20.0%)	19(40.4%)	
N +	19(16.7%)	10(31.3%)	2(20.0%)	3(12.0%)	4(8.5%)	
Nx	60(52.6%)	15(46.9%)	4(40.0%)	17(68.0%)	24(51.1%)	
LVI						0.653
No	87(76.3%)	25(78.1%)	6(60.0%)	19(76.0%)	37(78.7%)	
Yes	27(23.7%)	7(21.9%)	4(40.0%)	6(24.0%)	10(21.3%)	
Retroperitoneal relapse region						
Para-aortic lymph node	81 (71.1%)	28 (87.5%)	8(80.0%)	17(68.0%)	28(59.6%)	**0.046**
Common iliac lymph node	47 (41.2%)	4 (12.5%)	5(50.0%)	13 (52.0%)	25(53.2%)	**0.001**
Internal iliac lymph node	11 (9.6%)	0 (0%)	1(10.0%)	2 (8.0%)	8(17.0%)	**0.055**
External iliac lymph node	18 (15.8%)	1 (3.1%)	0(0%)	1 (4.0%)	16(34.0%)	**0.000**

The primary tumor sites included the renal pelvis (28.07%, 32/114), proximal ureter (8.77%, 10/114), middle ureter (21.93%, 25/114), and distal ureter (41.22%, 47/114). No statistically significant differences in baseline clinical characteristics were observed across patients with different primary tumor sites.

## Recurrence patterns and CTV delineation for tumors stratified by primary site

The extent of the radiotherapy target volume is critical for treatment efficacy. Historically, there has been no standardized consensus regarding prophylactic nodal irradiation fields for UTUC. The variation in lymph node dissection ranges based on primary tumor locations in UTUC suggests differences in lymphatic drainage patterns among different tumor sites. Therefore, analyzing the distribution patterns of postoperative nodal recurrences can inform optimal radiotherapy target delineation.The para-aortic region was the most frequent site of recurrence, which was observed in 81 patients (71.1%). The recurrence rate in this region was significantly greater in patients with renal pelvic and proximal ureteral tumors (UTUCs) than in those with middle and distal UTUCs (*P* = 0.046). The common iliac region was the second most common recurrence site, affecting 47 patients (41.2%). Compared with renal pelvic tumors, ureteral tumors were more likely to recur in the common iliac region (*P* = 0.001) (Table 1).

In renal pelvic UTUC, pelvic lymph node recurrence (LNR) is rare, with no cases observed in the internal iliac region and only 3% in the external iliac region. In contrast, distal ureteral tumors had higher recurrence rates in the internal (17%, *P* = 0.055) and external iliac regions (34.0%, *P* < 0.001) than other UTUC locations did (Table 1).

**Fig. 2:**
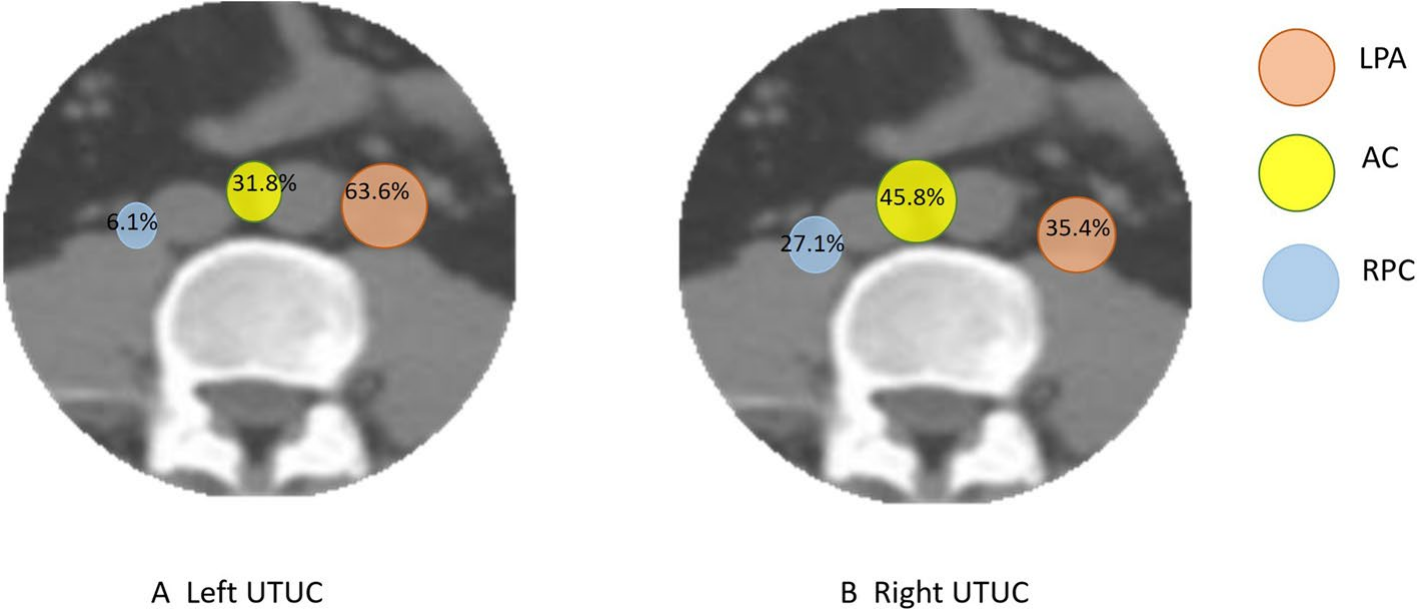
Recurrence lymph node site distribution ratio stratified by left UTUC and right UTUC

**Fig. 3:**
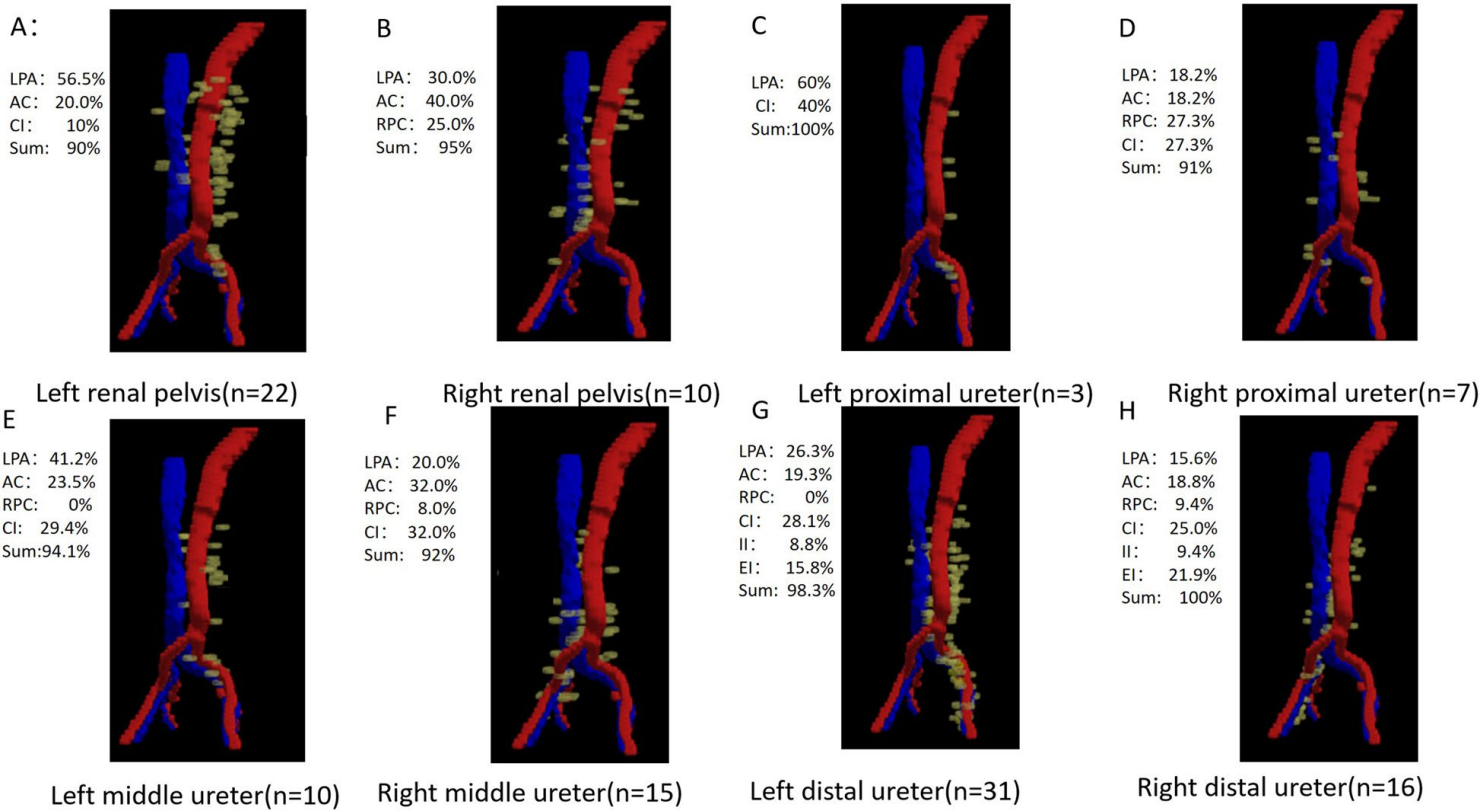
Recurrence lymph nodes distribution maps of UTUC by primary tumor location. **A** Left renal pelvis; **B** Right renal pelvis; **C** Left proximal ureter; **D** Right proximal ureter; **E** Left middle ureter; **F** Right middle ureter; **G** Left distal ureter; **H** Right distal ureter

Distinct laterality-based recurrence patterns were observed in the para-aortic region. Among the 48 right-sided UTUCs, the recurrence rates were comparable in the left para-aortic (LPA, 35.4%), aortocaval (AC, 45.8%), and right paracaval (RPC, 27.1%) regions (Fig. 2). In contrast, among the 66 left-sided UTUCs, recurrence events predominantly involved the LPA (63.6%) and AC (31.8%) regions, with only 6.1% occurring in the RPC region (*P* = 0.003) (Fig. 2). Figure 3 shows the distribution of abdominopelvic lymph node recurrence postoperatively in patients with different primary tumor locations. On the basis of this recurrence map, we designed site-specific radiotherapy clinical target volumes (CTVs) tailored to distinct primary tumor sites (Table 2). The CTV covered more than 90% of the recurrent lymph node regions.The radiotherapy target volumes stratified by primary tumor location in UTUC were more confined compared to uniform irradiation fields, which may further reduce radiation-induced toxicity. This study also presents the first precise delineation of nodal radiotherapy targets for UTUC based on anatomical mapping.

**Table 2:** Suggested boundaries of clinical target volume in UTUC with different primary locations

Subgroup	Cranial	Caudal	Abdominal Pelvic		Abdominal Pelvic		Medial		Lateral
							Left	Right	
Renal pelvic	bottom of T12	bifurcation of the common iliac	a 0.7-cm margin around aorta and inferior vena cava	-	anterior border of the vertebral body	-	Medial border of the inferior vena cava	a 1.0-cm margin around left para aorta	Renal fossa
Proximal ureter	bottom of T12	bifurcation of the common iliac	a 0.7-cm margin around aorta and inferior vena cava	-	anterior border of the vertebral body	-	Medial border of the inferior vena cava	a 1.0-cm margin around left para aorta	Medial of the psoas major muscle
Middle ureter	bottom of renal vessels	level of superior border of the femoral head	a 0.7-cm margin around aorta and inferior vena cava	a 0.7-cm margin around external iliac vessels	anterior border of the vertebral body	a 0.7-cm margin around internal iliac vessels	Medial border of the inferior vena cava	a 1.0-cm margin around left para aorta	Medial of the psoas major muscle
							a 0.7-cm margin around internal and external iliac (pelvic level)		
Distal ureter	bottom of renal vessels	2 cm below ureter-bladder Anastomosis	a 0.7-cm margin around aorta and inferior vena cava	a 0.7-cm margin around external iliac vessels	anterior border of the vertebral body	a 0.7-cm margin around internal iliac vessels	Medial border of the inferior vena cava	a 1.0-cm margin around left para aorta	Medial of the psoas major muscle
							a 0.7-cm margin around internal and external iliac (pelvic level)		

## Prognostic analysis

To assess outcomes in patients with isolated retroperitoneal lymph node metastasis (LNM), we excluded 12 patients with synchronous visceral metastases at diagnosis. The final cohort comprised 102 patients: 19 who received radiotherapy only (due to advanced age or poor performance status), 27 who received systemic therapy alone, and 56 who underwent combined treatment (systemic therapy and radiotherapy). There were no statistically significant differences in baseline characteristics between the groups (Supplementary Table 3). During a median follow-up of 22 months (range: 3–81), 60 patients (58.8%) developed distant metastases. The distribution of metastatic sites was as follows: 16 patients developed bone metastases, 14 patients developed liver metastases, 18 patients developed lung metastases, 1 patient developed psoas muscle metastases, and 11 patients developed supraclavicular or mediastinal lymph node metastases. Only three patients in the radiotherapy group developed local recurrence (4%) within the irradiated region.

**Fig. 4:**
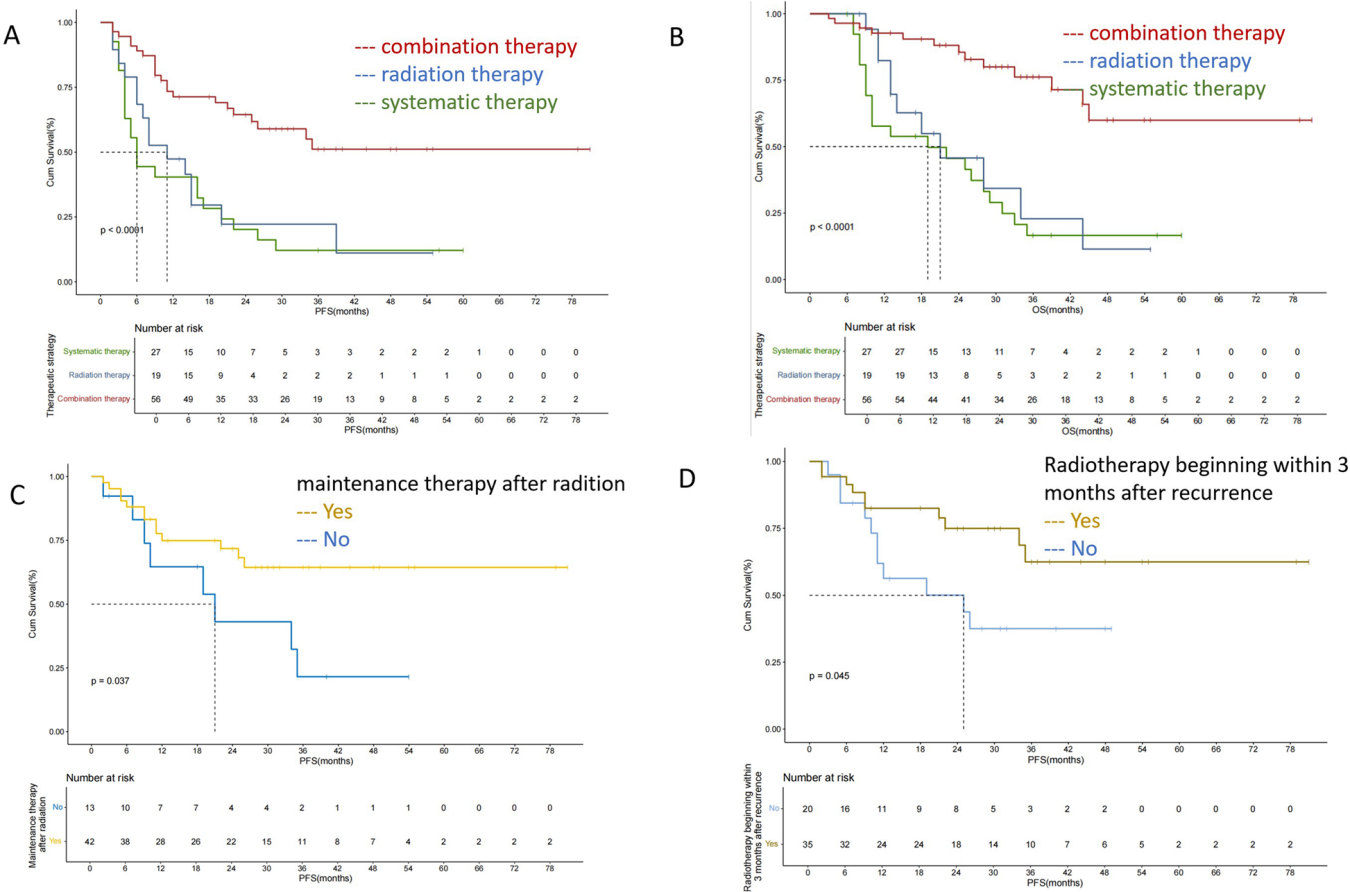
Kaplan–Meier curves for progression free survival (**A**), overall survival (**B**) of three treatment types;Kaplan–Meier curves for progression free survival of radiation therapy group with or without maintainace therapy (**C**), and radiation therapy beginning within or without 3 months (**D**)

Next, survival was compared between treatment groups to determine the optimal strategy.The 2-year PFS and OS rates for the entire cohort were 30.4% and 49.0%, respectively. The 2-year PFS rates for the combination therapy, radiation therapy and systemic therapy groups were 65.7%, 21.1% and 20.2%, respectively (*P* < 0.001). The 2-year OS rates for the combination therapy, radiation therapy and systemic therapy groups were 87.9%, 48.1% and 45.6%, respectively (*P* < 0.001).The combination therapy group demonstrated significantly improved clinical outcomes compared to monotherapy group (Fig. 4).

According to the univariate analysis, radiotherapy combined with systemic therapy, radiotherapy beginning within 3 months after LNM, maintenance therapy after radiation, and consolidation radiotherapy emerged as significant prognostic factors for PFS. Multivariate analysis further revealed that combination therapy (*p* = 0.043, hazard ratio (HR): 0.331 [0.114–0.996]), radiotherapy beginning within 3 months after recurrence (*p* = 0.002, HR: 0.340 [0.174–0.667]), and maintenance therapy after radiation (*p* = 0.039, HR: 0.359 [0.163–0.956]) were independent predictors of PFS after LNM. According to the univariate analysis, sex, combination therapy, maintenance therapy after radiation and distant metastasis emerged as significant prognostic factors of OS after LNM. Multivariate analysis further revealed that maintenance therapy after radiation (*p* = 0.023, hazard ratio (HR): 0.326 [0.123–0.859]) and distant metastasis (*p* = 0.003, HR: 6.780 [1.916–23.993]) were significant prognostic factors for OS after LNM (Tables 3 and 4).

**Table 3:** Univariate and multivariable analyses of the prognostic factors for progression free survival and overall survival time after retroperitoneal LNM

Subgroup	Univariate HR (95% CI) PFS	*P* value	Multivariate HR (95% CI) PFS	*P* value	Univariate HR (95% CI) OS	*P* value	Multivariate HR (95% CI) OS	*P* value
Age group								
< 70	Reference				Reference			
≥ 70	1.497(0.899–2.493)	0.121			1.580(0.880–2.838)	0.126		
Gender								
Female	Reference				Reference		Reference	
Male	1.402(0.844–2.328)	0.191			1.863(1.030–3.372)	**0.04**	1.424(0.625–3.244)	0.401
Multifocal tumor								
No	Reference				Reference			
Yes	0.845(0.415–1.719)	0.642			1.021(0.455–2.291)	0.960		
Tumor grade								
G2	Reference				Reference			
G3	0.926(0.492–1.744)	0.812			0.692(0.322–1.485)	0.344		
T stage								
T1-2	Reference				Reference			
T3-4	0.919(0.567–1.492)	0.733			0.964(0.489–1.899)	0.916		
LNM								
N0	Reference				Reference			
N +	0.562(0.245–1.286)	0.172			0.510(0.186–1.393)	0.189		
Nx	0.668(0.379–1.177)	0.163			0.607(0.322–1.143)	0.122		
LVI								
No	Reference				Reference			
Yes	1.020(0.530–1.962)	0.953			1.172(0.545–2.523)	0.684		
Adjuvant therapy								
No	Reference				Reference			
Yes	0.667(0.328–1.356)	0.263			0.390(0.140–1.090)	0.071		
Treatment type								
Systemic therapy	Reference		Reference		Reference		Reference	
Radiotherapy	0.889(0.468–1.69)	0.719	-		-	-	-	
Combination therapy	0.275(0.151–0.499)	**0.000**	0.331(0.114–0.996)	**0.043**	0.259(0.115-0.584)	**0.001**	0.741(0.277–1.985)	0.551
Radiotherapy time after recurrence								
≥ 3 months	Reference				Reference			
< 3 months	0.438(0.229–0.438)	**0.013**		**0.002**	0.509(0.228–1.138	0.10		
Maintenance therapy after Radiation								
No	Reference				Reference		Reference	
Yes	0.295(0.150–0.582)	**0.000**	0.395(0.163–0.956)	**0.039**	0.188(0.074–0.474)	**0.008**	0.326(0.123–0.859)	**0.023**
Radiotherapy type								
Consolidation	Reference		Reference		Reference			
Salvage	3.637(1.484–8.910)	**0.005**	1.995(0.690–5.766)	0.202	1.403(0.471–4.181)	0.543		
Distant metastasis								
No	-	**-**			Reference		Reference	
Yes	-	**-**			15.515(4.769–50.47)	**0.000**	6.780(1.916–23.993)	**0.003**
Metastasis site	-	**-**						
liver	-	**-**			Reference			
bone	-	**-**			0.571(0.231–1.411)	0.224		

**Table 4:** Multivariable analyses of the prognostic factors for progression free survival and overall survival time after retroperitoneal LNM

Variables	Risk Factor	HR (95% CI)	*P* value
PFS			
Treatment type	Combination Vs Systemic therapy	0.331(0.114–0.996)	**0.043**
Radiotherapy time after recurrence	< 3 month Vs ≥ 3 month	0.340(0.174–0.667)	**0.002**
Maintenance therapy after Radiation	Yes Vs No	0.395(0.163–0.956)	**0.039**
Radiotherapy type	Salvage Vs Consolidation	1.995(0.690–5.766)	0.202
CSS			
Gender	Male Vs Female	1.079(0.421–2.767)	0.874
Radiotherapy time after recurrence	< 3 month Vs ≥ 3 month	0.478(0.202–1.129)	0.092
Treatment type	Radiation Vs Systemic therapy	0.741(0.277–1.985)	0.551
Maintenance therapy after Radiation	Yes Vs No	0.248(0.090–0.628)	**0.007**
Distant metastasis	Yes Vs No	5.941(1.697–20.802)	**0.005**

## Adverse events

The incidence rates of grade 1/2 leukopenia, grade 1/2 acute gastrointestinal disorders, and grade 1/2 thrombocytopenia in the combination therapy group were 12.7%/21.8%, 41.8%/21.8%, and 5.5%/5.5%, respectively. Only one patient had grade 3 leukopenia and thrombocytopenia (Supplementary Table 2).The findings suggest that radiotherapy combined with systemic pharmacotherapy was well-tolerated without significantly increasing treatment-related toxicities.

## Discussion

UTUC is a subtype of urothelial carcinoma with poor prognosis. After unilateral nephrectomy, patients often experience impaired renal function, and once postoperative recurrence or metastasis occurs, the efficacy of pharmacological treatment is suboptimal, resulting in low survival rates for metastatic UTUC. Postoperative cN + represents a distinct metastatic state. Our findings suggest that combining radiotherapy with systemic therapy can further improve patient outcomes compared to pharmacotherapy alone. Subgroup analysis revealed that patients who initiated radiotherapy within 3 months of recurrence and received subsequent maintenance immunotherapy derived greater clinical benefit.

Metastatic urothelial carcinoma generally has a poor prognosis, clinically, node-positive (cN +) UTUC is also classified as metastatic disease[4]. In a previous study, patients with visceral metastases had significantly worse outcomes than those with lymph node metastases alone[13]. Metastasis only to the lymph nodes is a prognostic factor for metastatic urothelial carcinoma after platinum-based therapy[14]. EAU guidelines recognize lymph node-only metastasis as a distinct metastatic pattern for UTUC. According to current guidelines, RNU with template-based LND may be considered through multidisciplinary discussion for patients who exhibit a positive response to initial systemic therapy[4]. However, the optimal LND template varies significantly depending on the tumor location, and complete dissection remains technically challenging due to the high risk of postoperative complications[15]. Consequently, the complete LND rate in clinical practice remains suboptimal (approximately 15–30%), and the lack of complete LND often results in inaccurate nodal staging and contributes to lymph node recurrence, which is the predominant type of metastasis observed following UTUC resection[13, 16].

Urothelial carcinoma is radiosensitive, making radiotherapy a viable local treatment option. In our previous study, radiotherapy alone achieved a favorable objective response rate in patients with unresectable UTUC[17, 18], indicating that UTUC is radiosensitive. Furthermore, for patients with extensively metastatic UTUC, the combination of radiotherapy with systemic therapy demonstrated improved progression-free survival (PFS) compared to systemic therapy alone[19]. For bladder cancer, consolidative chemoradiotherapy has improved OS and PFS in patients with pelvic or retroperitoneal lymph node metastases[8]. Similarly, metastasis-directed radiation therapy has proven safe and well tolerated following radical cystectomy[20]. This study represents the first investigation evaluating the efficacy and safety of radiotherapy combined with systemic therapy for postoperative lymph node metastasis in UTUC. The results demonstrate that the combined modality significantly improves patient outcomes compared to systemic therapy alone, with earlier initiation of radiotherapy (within 3 months after starting pharmacotherapy) associated with greater clinical benefit.

The 2-year PFS of patients receiving systemic therapy was 20.2% in our study, which was higher than that of mUC patients receiving chemotherapy in other studies[7]. Our findings indicate that patients with retroperitoneal lymph node metastasis (RPLNM) alone exhibit better response to systemic therapy compared to those with extensively metastatic UTUC. Since chemotherapy use impairs renal function, 20 patients in this study received radiotherapy alone. These patients also achieved a 2-year PFS of 21.1%, which is similar to that of patients treated with systemic therapy (20.2%). This finding aligns with previous reports demonstrating the efficacy of radiotherapy in controlling local metastases and palliating symptoms in patients with metastatic urothelial carcinoma[18, 21]. Multivariate analysis identified combination therapy and postradiation maintenance therapy as independent predictors of PFS, suggesting that integrating local consolidative therapy with systemic treatment yields superior outcomes to systemic therapy alone for retroperitoneal LNM.Moreover, our study revealed that maintenance therapy following radiation significantly correlates with both PFS and OS. These findings corroborate Andre's finding of superior OS with concurrent systemic therapy and radiation versus radiation alone[20]. Kang's study further supports this approach, as the longest median OS (19 months) was observed in the group with concurrent chemoradiotherapy plus immunotherapy[13]. The timing of local therapy is equally critical: our prior research demonstrated longer PFS when radiotherapy was initiated before first-line chemotherapy failure than when it was initiated after treatment failure[19]. In the current study, early intervention (within 3 months of recurrence) similarly improved PFS. Remarkably, the median OS has not yet been reached in our cohort, suggesting that combination therapy may have curative outcomes in select patients.

The grade ≥ 3 adverse events included peripheral sensory neuropathy, fatigue, hyperglycemia, diarrhea, and neutropenia in ADC therapies[7, 11]. The chemotherapy group predominantly exhibited grade ≥ 3 toxicities of fatigue, anemia, neutropenia, and thrombocytopenia[7].Notably, the incidence of grade ≥ 3 adverse events during combination therapy was remarkably low, with the majority of patients experiencing only grade 1–2 gastrointestinal reactions.These findings suggest that radiotherapy does not appear to augment the toxicity profile of existing systemic therapies, demonstrating favorable tolerability when used in combination.

The recent advent of immune checkpoint inhibitors (ICIs) and antibody‒drug conjugates (ADCs) has expanded treatment options for patients with renal impairment. Given the known immunomodulatory effects of radiotherapy, the potential synergy between ICIs and radiotherapy in patients with metastatic UC warrants further investigation[22]. Previous studies have demonstrated favorable safety profiles for radiotherapy combined with immunotherapy in metastatic urothelial carcinoma. While data on radiotherapy plus antibody–drug conjugates (ADCs) remain limited in this setting, evidence from breast cancer suggests good tolerability of this combination approach[23].

The extent of the CTV is directly related to the local control rate of radiotherapy. However, the use of the clinical target volume (CTV) for UTUC radiotherapy has been controversial in previous studies[24, 25]. A retrospective study revealed that the radiotherapy target volume covered only 70% of recurrent retroperitoneal lesions[26]. Our retrospective study revealed distinct patterns of lymph node recurrence in UTUC based on primary tumor location[6]. This prospective study further confirms that the anatomical distribution of postoperative lymph node metastases correlates with the primary tumor sites.In our research, renal pelvis tumors and proximal ureter tumors rarely recurred below the common iliac level. Patients with distal ureteral carcinoma demonstrate elevated recurrence risks in both abdominal and pelvic lymph node regions.This finding is consistent with the metastatic patterns observed during lymph node dissection in previous UTUC studies[27].In this study, we designed radiotherapy target delineation on the basis of high-recurrence regions, achieving a local recurrence-free survival (LRFS) rate of 96%. To our knowledge, this is the first proposal for delineating radiotherapy target areas in the abdominal and pelvic lymph node drainage regions for UTUC.

## Conclusions

Radiotherapy combined with systemic therapy improved survival PFS with tolerable adverse effects for UTUC patients with rLNM. We also provide a method for radiotherapy target volume delineation on the basis of the high-recurrence regions.This lays the groundwork for future prospective studies evaluating radiotherapy combined with systemic therapy.

## Supplementary Information

Supplementary Material 1: Table 1. Local recurrence lymph nodes number of UTUC with different primary tumor locations. Table 2. Treatment-Related Adverse Events.

Supplementary Material 2: Table 3. Characteristics of 102 UTUC patients with positive lymph nodes and univariate analysis of prognostic factors.

## Data Availability

Data is provided within the manuscript or supplementary information files.

## Abbreviations

UTUC: Upper tract urothelial carcinoma
ACT: Adjuvant chemotherapy
LNR: Lymph node recurrence
OS: Overall survival
LRFS: Local recurrence-free survival
PFS: Progression-free survival
rLNM: Retroperitoneal lymph node metastasis
RNU: Radical nephroureterectomy
EAU: European Association of Urology
LPA: Left lateral para-aortic
AC: Aortocaval
RPC: Right paracaval
PET: Positron emission tomography
GTV: Gross tumor volume
CTV: Clinical target volume
EV: Enfortumab vedotin
DV: Disitamab vedotin
ICI: Immune checkpoint inhibitor
ADC: Antibody‒drug conjugate vedotin
