# Supplementary material for: Efficacy of radiotherapy combined with systemic therapy for retroperitoneal lymph node metastasis in upper tract urothelial carcinoma(UTUC) patients after radical nephroureterectomy
Source: World J Surg Oncol. 2025 Nov 11;23:428. doi: 10.1186/s12957-025-03943-7 (PMC12607141; doi:10.1186/s12957-025-03943-7)
Supplement: Supplementary file 1 — Supplementary Material 1: Table 1. Local recurrence lymph nodes number of UTUC with different primary tumor locations. Table 2. Treatment-Related Adverse Events. [file 12957_2025_3943_MOESM1_ESM.docx]

**Supplementary Table 1 Local recurrence lymph nodes number of UTUC with different primary tumor locations**

| Local relapse region | All lymph nodes  N=302 | Renal Pelvis  N=99 | Proximal ureter  N=20 | Middle ureter  N=65 | Distal ureter  N=138 |
| --- | --- | --- | --- | --- | --- |
| Para-aortic | 212(70.2%) | 83(83.8%) | 13(65.0%) | 37(56.9%) | 79(55.6%) |
| Common iliac | 57(18.9%) | 5(5.1%) | 6(30.0%) | 15(23.2%) | 31(21.8%) |
| Internal iliac | 11(3.6%) | 0(0%) | 1(5.0%) | 2(3.1%) | 8(5.6%) |
| External iliac | 22(7.3%) | 1(1.0%) | 0(0%) | 1(1.5%) | 20(14.1%) |

**supplementary table2 Treatment-Related Adverse Events**

| Toxicity | Grade 1, *n* (%) | Grade 2, *n* (%) | Grade 3, *n* (%) |
| --- | --- | --- | --- |
| Gastrointestinal | 23 (41.8%) | 12 (21.8%) | 0 (0) |
| Leukopenia | 7 (12.7%) | 12 (21.8%) | 1 (1.8%) |
| Thrombocytopenia | 3 (5.5%) | 3 (5.5%) | 1 (1.8%) |
