# Supplementary material for: Efficacy of radiotherapy combined with systemic therapy for retroperitoneal lymph node metastasis in upper tract urothelial carcinoma(UTUC) patients after radical nephroureterectomy
Source: World J Surg Oncol. 2025 Nov 11;23:428. doi: 10.1186/s12957-025-03943-7 (PMC12607141; doi:10.1186/s12957-025-03943-7)
Supplement: Supplementary file 2 — Supplementary Material 2: Table 3. Characteristics of 102 UTUC patients with positive lymph nodes and univariate analysis of prognostic factors. [file 12957_2025_3943_MOESM2_ESM.docx]

**Supplementary Table 3 Characteristics of 102 UTUC patients with positive lymph nodes and univariate analysis of prognostic factors**

| Characters | All patients  N=102 | Radiotherapy  N=19 | systemic therapy  N=27 | combined treatment  N=56 | P value |
| --- | --- | --- | --- | --- | --- |
| Age group |  |  |  |  |  |
| ＜70 | 61(53.5%) | 9(47.4%) | 18(66.7%) | 34(60.7%) | 0.413 |
| ≥70 | 41(46.5%) | 10(52.6%) | 9(33.3%) | 22(39.3%) |  |
| Gender |  |  |  |  |  |
| Female | 47(53.9%) | 11(57.9%) | 11(40.7%) | 33(58.9%) | 0.276 |
| Male | 55(46.1%) | 8(42.1%) | 16(59.3%) | 23(41.1%) |  |
| Multifocal tumor |  |  |  |  | 0.326 |
| No | 85(83.2%) | 18(94.7%) | 21(80.8%) | 45(86.4%) |  |
| Yes | 17(16.8%) | 1(5.3%) | 5(19.2%) | 11(19.6%) |  |
| Tumor grade |  |  |  |  | 0.970 |
| G2 | 18(17.6%) | 3(15.8%) | 5(18.5%) | 10(17.9%) |  |
| G3 | 84(82.4%) | 16(84.2%) | 22(81.5%) | 46(82.1%) |  |
| T stage |  |  |  |  | 0.822 |
| T1 | 9(8.8%) | 3(15.8%) | 2(7.4%) | 4(7.1%) |  |
| T2 | 33(32.4%) | 7(36.8%) | 10(37.0%) | 16(28.6%) |  |
| T3 | 54(52.9%) | 8(42.1%) | 13(48.1%) | 33(58.9%) |  |
| T4 | 4(5.9%) | 1(5.3%) | 2(7.4%) | 3(5.4%) |  |
| LNM |  |  |  |  | 0.136 |
| N0 | 28(27.5%) | 6(31.6%) | 11(40.7%) | 11(19.6%) |  |
| N+ | 17(16.7%) | 1(5.3%) | 3(11.1%) | 13(23.2%) |  |
| Nx | 57(55.9%) | 12(63.2%) | 13(48.1%) | 32(57.1%) |  |
| LVI |  |  |  |  | 0.328 |
| No | 82(80.4%) | 13(68.4%) | 23(85.2%) | 46(82.1%) |  |
| Yes | 20(19.6%) | 6(31.6%) | 4(14.8%) | 10(17.9%) |  |
